# *Notes from the Field:* Toxigenic *Corynebacterium ulcerans* in Humans and Household Pets — Utah and Colorado, 2022–2023

**DOI:** 10.15585/mmwr.mm7323a3

**Published:** 2024-06-13

**Authors:** Amanda R. Metz, Angie White, Jared Ripplinger, Emily Spence Davizon, Meghan Barnes, Matt Bauer, Lauren Butler, Natalie S. Marzec, Shannon R. Matzinger, Valerie Bampoe, Hong Ju, Ingrid C. McCall, Marissa Fraire, Yanhui Peng, Willy Lanier

**Affiliations:** ^1^Colorado Department of Public Health and Environment; ^2^Bear River Health Department, Logan, Utah; ^3^Utah Department of Health and Human Services; ^4^Larimer County Department of Health, Larimer, Colorado; ^5^Division of Bacterial Diseases, National Center for Immunization and Respiratory Diseases, CDC; ^6^Career Epidemiology Field Officer Program, CDC.

**Summary**

**What is already known about this topic?**

Toxigenic *Corynebacterium ulcerans* can cause diphtheria-like illness in humans. Transmission of this uncommon zoonotic pathogen between humans and animals is poorly understood.

**What is added by this report?**

Investigations in Utah and Colorado provide evidence of the risk for *C. ulcerans* transmission between humans and household pets. Treatment based on antibiotic susceptibility testing results led to successful infection control.

**What are the implications for public health practice?**

A One Health (human, animal, and environmental health) approach can be used to control the transmission of and infection with *C. ulcerans*.

Toxigenic *Corynebacterium ulcerans*, an uncommon zoonotic pathogen, can cause diphtheria-like illness in humans. In April 2022, the Utah Department of Health and Human Services was notified of laboratory-confirmed toxigenic *C. ulcerans* isolated from a nonhealing leg wound of a Utah resident with diabetes, and in April 2023, the Colorado Department of Public Health and Environment was notified of laboratory-confirmed toxigenic *C. ulcerans* isolated from a Colorado resident experiencing nonresolving upper respiratory symptoms. Health officials in Utah and Colorado investigated these infections in humans and their household pets.* A One Health approach^†^ could be considered to control the transmission and infection of *C. ulcerans*. This activity was reviewed by CDC, deemed not research, and was conducted consistent with applicable federal law and CDC policy.^§^

## Investigation and Outcomes

### Utah Case Investigation

The Utah resident lived with a spouse, another housemate, three cats, and one dog. Health officials recommended wound covering, masking, and use of disinfectant on household surfaces. The patient’s spouse and the other housemate and the four pets (all asymptomatic) were tested; toxigenic *C. ulcerans* was isolated from the patient’s spouse and two cats. Whole genome sequencing (WGS) of isolates from these cats and the index patient found that the isolates were the same type.^¶^ The index patient, the patient’s spouse, and the other housemate were treated empirically with penicillin (*1*), and the four pets were treated with amoxicillin and clavulanic acid. After treatment, *C*. *ulcerans* was isolated from the index patient and all pets but not from the spouse; the other housemate was not retested. Subsequently, antibiotic susceptibility results from the index patient’s initial isolate indicated that the organism was susceptible to erythromycin but moderately susceptible to penicillin, indicating a higher dose of penicillin would be necessary.** After the index patient, the patient’s spouse, the housemate, and the four pets were treated with erythromycin, testing for all persons and pets living in the house did not yield *C. ulcerans*.

### Colorado Case Investigation

The Colorado patient reported close contact with a spouse, two dogs living at the same house, and a visiting family member and dog who were at the house for two nights; all human and animal contacts were asymptomatic. Health officials recommended that the patient stay home from work and wear a mask during activities outside the home. *C*. *ulcerans* was isolated from the patient and the visiting dog^††^ but not from the visiting family member and the patient’s own two dogs. Isolates from the patient and visiting dog were of the same WGS type.^§§^ The patient’s spouse declined testing but was treated empirically with erythromycin. During treatment, antibiotic susceptibility testing results for both the human and dog isolates indicated susceptibility to erythromycin. After treatment with erythromycin, follow-up testing for the patient and the visiting dog did not yield *C. ulcerans*.

## Preliminary Conclusions and Actions

Toxigenic *Corynebacterium diphtheriae* infection is nationally notifiable; however, infection with toxigenic *C. ulcerans* is not. Illness caused by toxigenic *C*. *ulcerans* can mimic toxigenic *C*. *diphtheriae* infection and necessitates prompt identification, treatment, and control (*2*). In addition, surveillance and routine vaccination with diphtheria toxoid-containing vaccines are important to protect persons from severe toxin-mediated illness caused by toxigenic *Corynebacterium* spp. (*3*).

These Utah and Colorado cases represent the first reported U.S. cases of toxigenic *C*. *ulcerans* infection among humans with concurrent household pet colonization (*4**,**5*). *C*. *ulcerans* is believed to be zoonotic (*5*); human-to-human transmission has not been documented. Evidence from this investigation suggests that transmission of toxigenic *C*. *ulcerans* between humans and household pets occurred, although the direction of transmission could not be determined.

Although penicillin or erythromycin are recommended treatments for toxigenic *Corynebacterium* infections (*1*), this investigation suggests that treatment of both human and veterinary patients should be based on antibiotic susceptibility results. Health officials can utilize a One Health approach considering human, animal, and environmental health to control *C. ulcerans* transmission and infections.

## Footnotes

* Wound, oropharyngeal, or nasal swabs were collected from human patients. Oral, nasal, or rectal swabs were collected from pets. Index patients’ initial swabs were tested via culture and identified using matrix-assisted laser desorption/ionization time-of-flight. CDC tested specimens by culture and polymerase chain reaction; positive specimens were then tested by Elek immunoprecipitation assay (https://www.cdc.gov/diphtheria/php/laboratories/index.html) to detect diphtheriae toxin. For these investigations, a case was defined as an infection of a human or animal with *C. ulcerans*.

†
https://www.cdc.gov/one-health/about/index.html

§ 45 C.F.R. part 46.102(l)(2), 21 C.F.R. part 56; 42 U.S.C. Sect. 241(d); 5 U.S.C. Sect. 552a; 44 U.S.C. Sect. 3501 et seq.

¶ The WGS sequence types (ST) for Utah and Colorado strains were ST325 and ST699, respectively.

** Susceptibility testing of Utah’s index patient’s initial isolate was conducted by a commercial laboratory.

†† Health officials recommended that the visiting dog be isolated from other humans and animals until after receipt of single set of negative test results.

§§ CDC performed antibiotic susceptibility testing for Colorado’s investigation.
